# 纯化供者CD34^+^细胞输注治疗单倍体造血干细胞移植后难治性造血重建不良8例临床分析

**DOI:** 10.3760/cma.j.issn.0253-2727.2023.12.010

**Published:** 2023-12

**Authors:** 云 何, 瑞 马, 慧芳 王, 圆圆 张, 萌 吕, 晓冬 莫, 晨华 闫, 昱 王, 晓辉 张, 兰平 许, 开彦 刘, 晓军 黄, 于谦 孙

**Affiliations:** 北京大学人民医院，北京大学血液病研究所，国家血液系统疾病临床医学研究中心，造血干细胞移植北京市重点实验室，北京 100044 Peking University People's Hospital, Peking University Institute of Hematology, National Clinical Research Center for Hematologic Disease, Beijing Key Laboratory of Hematopoietic Stem Cell Transplantation, Beijing 100044, China

造血重建不良（poor hematopoietic reconstitution, PHR）是异基因造血干细胞移植（allo-HSCT）的严重并发症，表现为植入功能不良（poor graft function, PGF）或持续性血小板减少（prolonged isolated thrombocytopenia, PT）[1]。文献报道PGF的发生率为5％～27％[2]，原发PT发生率为10.1％[3]。PGF及PT均可导致非复发死亡增加，严重影响移植预后[4]–[6]。PHR目前尚无标准治疗办法，常用治疗手段包括输血支持、二次移植、供者淋巴细胞输注（DLI）和促造血生长因子等，但输血支持伴随输注相关铁过载等不良反应，二次移植或DLI可致非复发死亡率升高和GVHD发生。目前allo-HSCT后PHR的发生机制尚未阐明，可能与CD34^+^细胞数量或质量受损有关[1],[7]–[10]。再次输注纯化供者CD34^+^细胞可促进患者造血干/祖细胞重建，国外文献报道治疗PHR的有效率为58.3％～100％[11]–[19]。本研究对近年来在本中心接受纯化供者CD34^+^输注的8例单倍体造血干细胞移植后难治性PHR患者进行回顾性分析。

## 病例与方法

一、病例

本研究纳入2019年1月至2022年4月在北京大学人民医院8例接受纯化供者CD34^+^细胞输注的单倍体造血干细胞移植后难治性PHR患者。

二、定义

PGF：移植28 d后仍存在中性粒细胞缺乏（ANC<0.5×10^9^/L）及血小板减少（PLT<20×10^9^/L或血小板输注依赖）伴或不伴血红蛋白下降并持续2周以上，嵌合率完全供者型，排除复发、严重GVHD、感染以及药物相关骨髓抑制[3]。PGF分为原发性和继发性，继发性PGF是指植入后再次出现至少两系血细胞计数下降。

PT：移植后60 d嵌合度为完全供者型，但血小板计数仍<20×10^9^/L或未脱离血小板输注依赖，而粒系（ANC>0.5×10^9^/L且脱离G-CSF）及红系（HGB≥70 g/L并脱离红细胞输注）恢复良好[5]。

三、供者CD34^+^细胞采集、分选与输注

供者采用粒细胞集落刺激因子（G-CSF）5 µg/kg进行动员，动员至第4日时采集供者外周血干细胞。应用CliniMACS Plus系统对采集的造血干细胞进行CD34^+^细胞分选，流式细胞术测定CD34^+^细胞，活细胞染料DRAQ测定分选后细胞活性。分选后的细胞存于4 °C冰箱，于24 h内输注完毕，输注前予葡萄糖酸钙注射液抗过敏。

四、纯化供者CD34^+^细胞输注疗效评估

造血恢复：ANC≥0.5×10^9^/L并脱离G-CSF，HGB≥70 g/L并脱离红细胞输注，PLT≥20×10^9^/L连续7 d并脱离血小板输注。PGF治疗有效：输注纯化供者CD34^+^细胞后三系（红细胞、白细胞及血小板）造血恢复；PGF治疗无效：输注纯化供者CD34^+^细胞后三系均未恢复。PT治疗有效：输注纯化供者CD34^+^细胞后血小板恢复。

五、随访及统计学处理

所有患者均通过门诊或电话随访至2022年4月30日。OS时间为输注纯化供者CD34^+^细胞到随访截止或死亡的时间。计量资料用“中位数（范围）”表示。

## 结果

一、患者一般资料

8例难治性PHR患者中继发性PGF 5例，原发性PT 3例；女3例，男5例，中位年龄31.5（18～65）岁。再生障碍性贫血2例，骨髓增生异常综合征3例，急性B淋巴细胞白血病2例，急性髓系白血病1例。所有患者接受清髓性预处理。输注MNC中位数为10.47（6.83～17.35）×10^8^/kg，CD34^+^细胞中位数为2.30（0.41～4.93）×10^6^/kg。

纯化供者CD34^+^细胞输注前，8例患者均有巨细胞病毒（CMV）感染史，其中4例为难治性CMV血症；4例患者曾发生Ⅱ～Ⅳ度急性GVHD。详见表1。

**表1:** 8例接受纯化供者CD34^+^细胞输注单倍体造血干细胞移植后造血重建不良（PHR）患者的临床资料

例号	性别	年龄	原发病	移植相关资料						
				供患者关系	供患者血型	DSA	预处理方案	GVHD预防方案	移植物	MNC输注量（×10^8^/kg）
1	男	32	AML（CR1）	父供子	B+供B+	阴性	Bu-Cy+rATG	MTX+MMF+CsA	BM+PB	6.83
2	女	31	SAA	父供女	B+供AB+	阳性	Bu-Cy+rATG	MTX+MMF+CsA	BM+PB	14.16
3	女	65	B-ALL（Mll-AF4阳性，CR2）	子供母	O+供A+	阴性	Bu-Flu/Cy+rATG	MTX+MMF+CsA	PB	11.05
4	男	41	MDS-EB1	父供子	O+供O+	阴性	Bu-Flu/Cy+rATG	MTX+MMF+CsA	BM+PB	10.05
5	男	58	B-ALL（CR1，移植前MRD阳性）	兄供弟	A+供A+	阴性	Bu-Flu/Cy+rATG	MTX+MMF+CsA	PB	9.85
6	男	29	SAA-II	母供子	O+供O+	阴性	Bu-Flu/Cy+rATG	MTX+MMF+CsA	BM+PB	17.35
7	男	18	MDS-EB2	父供子	A+供A+	阴性	Bu-Cy+rATG	MTX+MMF+CsA	PB	7.82
8	女	20	MDS-EB2	父供女	B+供B+	阴性	Bu-Cy+rATG	MTX+MMF+CsA	PB	10.89

例号	性别	年龄	移植相关资料				
			CD34^+^细胞输注量（×10^6^/kg）	粒细胞植活时间（d）	血小板植活时间（d）	移植后合并症	嵌合度
1	男	32	0.41	16	40	急性GVHD（皮肤肠道），CMV血症	完全供者型
2	女	31	1.61	12	21	急性GVHD（皮肤、肝脏、消化道），CMV血症、HC	完全供者型
3	女	65	2.36	20	43	横断性脊髓炎，癫痫大发作，肺部感染（耐碳青霉烯铜绿），光滑念珠菌），CMV血症，菌血症（铜绿、屎肠球）	完全供者型
4	男	41	2.09	22	40	难治性CMV血症，EBV血症，腺病毒血症，HC，血播散肺结核	完全供者型
5	男	58	2.41	13	10	难治性CMV血症，病毒性脑炎（HHV-6），难治性EBV血症，TMA，HC，呼吸衰竭，病毒性肺炎（CMV、HHV-6），病毒性心肌炎	完全供者型
6	男	29	3.54	11	/	急、性GVHD（皮肤），难治性CMV血症	完全供者型
7	男	18	2.24	12	/	HC，CMV血症	完全供者型
8	女	20	4.93	15	/	难治性CMV血症，急性GVHD（皮肤），消化道出血，肺部感染（CMV、小包根霉）	完全供者型

注 AML：急性髓系白血病；B-ALL：急性B淋巴细胞白血病；MDS-EB1：骨髓增生异常综合征伴原始细胞过多1型；MDS-EB2：骨髓增生异常综合征伴原始细胞过多2型；SAA：重型再生障碍性贫血；CR1：第1次完全缓解；CR2：第2次完全缓解：FCM：流式细胞术；MRD：微小残留病；DSA：供者特异性抗体；PB：外周干细胞；BM：骨髓；Bu：白消安；Cy：环磷酰胺；Flu：氟达拉滨；rATG：兔抗人胸腺细胞免疫球蛋白；MTX：甲氨蝶呤；MMF：霉酚酸酯；CsA：环孢素A；TMA：血栓性微血管病；HC：出血性膀胱炎；CMV：巨细胞病毒；EBV：EB病毒；HHV-6：人类疱疹病毒6型；/：不适用

二、造血不良情况

8例PHR患者输注纯化供者CD34^+^细胞时均为完全供者型，骨髓增生Ⅲ级2例，增生减低6例，7例患者骨髓穿刺涂片未见巨核细胞。

所有8例患者诊断PHR后接受了包括糖皮质激素、TPO-RA、MSC在内的多种促造血治疗，疗效不佳。5例PGF患者诊断PGF中位时间为移植后75（63～129）d，诊断PGF至输注纯化供者CD34^+^细胞的中位间隔时间为90（37～109）d，3例PT患者造血干细胞移植至纯化供者CD34^+^细胞输注中位时间为161（131～239）d。

三、纯化供者CD34^+^细胞输注情况

8例患者纯化供者CD34^+^细胞中位输注量为1.74（1.03～3.28）×10^6^/kg（表2）。

**表2:** 8例单倍体造血干细胞移植后造血重建不良（PHR）患者纯化供者CD34^+^细胞输注情况、疗效及随访结局

例号	PHR类型	诊断PHR时间（d）	输注时间（d）	输注量（×10^6^/kg）	输注时骨髓象及MRD	输注时GVHD预防
1	继发性PGF	+75	+162	1.54	骨髓增生Ⅲ级，原始细胞1%，巨核细胞8个；FCM阴性；WT1 0.32%	无
2	继发性PGF	+63	+172	1.03	骨髓增生Ⅴ级，未见巨核细胞	无
3	继发性PGF	+63	+153	1.69	骨髓增生Ⅴ级，未见巨核细胞；FCM阴性；MLL AF4阴性	无
4	继发性PGF	+129	+228	1.30	骨髓增生Ⅳ级，未见巨核细胞；FCM阴性；WT1 0.11%	无
5	继发性PGF	+94	+131	3.28	骨髓增生Ⅴ级，未见巨核细胞；FCM阴性	无
6	原发性PT	/	+161	1.79	骨髓增生Ⅲ级，未见巨核细胞；造血容量<5%	CsA
7	原发性PT	/	+131	2.69	骨髓增生Ⅴ级，未见原始、巨核细胞；FCM阴性；WT1 0.067%	CsA
8	原发性PT	/	+239	3.07	骨髓增生Ⅴ级，未见巨核细胞；FCM阴性；WT1 0.083%	卢克替尼、他克莫司、糖皮质激素

例号	输注时合并症	PHR合并治疗	起效时间（d）	疗效评估	输注后合并症	结局
1	肺部真菌感染	十一酸睾酮，乙酰半胱氨酸，艾曲泊帕	16	有效	慢性GVHD（BOS）	生存
2	无	维甲酸，TPO-RA，达那唑	85	有效	慢性GVHD（皮肤2级）	生存
3	肺部感染、呼吸衰竭、横断性脊髓炎	十一酸睾酮，乙酰半胱氨酸，TPO-RA	/	无效	/	死亡
4	无	十一酸睾酮，乙酰半胱氨酸，TPO-RA，糖皮质激素	48	有效	无	生存
5	EBV、病毒性脑炎、细菌感染	MSC，乙酰半胱氨酸	/	/	/	死亡
6	无	MSC，TPO-RA，十一酸睾酮，乙酰半胱氨酸	20	有效	无	生存
7	无	TPO-RA，糖皮质激素，乙酰半胱氨酸	22	有效	无	生存
8	CMV血症、肺部感染、皮肤GVHD	MSC，糖皮质激素，TPO-RA	/	无效	/	肺部感染死亡

注 PGF：植入功能不良；PT：持续性血小板减少；GVHD：移植物抗宿主病；BOS：闭塞性细支气管炎综合征；EBV：EB病毒；CMV：巨细胞病毒；TPO-RA：血小板生成素受体激动剂；MSC：间充质干细胞；/：不适用

输注纯化供者CD34^+^细胞时，有3例患者仍在接受包括钙调磷酸酶抑制剂（CNI）在内的基础免疫抑制剂治疗，其余5例无基础免疫抑制剂治疗；4例患者合并感染。输注CD34^+^细胞时8例患者均无明显活动性急慢性GVHD。所有患者均未改变原有抗GVHD及其他原有治疗方案，输注前仅给予葡萄糖酸钙注射液抗过敏治疗，所有患者输注过程顺利，未发生特殊输注相关不良反应。

四、纯化供者CD34^+^细胞输注的疗效

8例患者中，1例患者无法评估疗效，其余7例患者中5例（71.42％）判定为有效，中位起效时间为22（16～85）d。

五、生存

随访至2022年4月5日，8例患者中5例存活，均为造血恢复患者。5例存活患者输注纯化CD34^+^细胞后均无新发感染或原有感染加重，未发生输注相关急性GVHD。长期随访中2例患者发生慢性GVHD（例1闭塞性细支气管炎综合征，例2皮肤2级慢性GVHD）。

## 讨论

CD34^+^细胞受损是PHR的可能机制之一[1],[7]–[9]，也是输注纯化CD34^+^细胞的理论基础。导致其发生的因素包括原发病状态、CD34^+^细胞数量、CMV血症、GVHD、供者特异性抗体（DSA）阳性、铁过载等[10],[20]。部分PHR患者对两种或三种以上促造血治疗无效，这些难治患者的造血重建是亟需解决的难题。

费新红等[22]应用输注纯化供者CD34^+^细胞治疗12例单倍型造血干细胞移植后移植物功能不良患者，10例获得完全缓解，1例获得部分缓解，1例无效。李智慧等[21]应用输注纯化供者CD34^+^细胞治疗10例allo-HSCT后移植物功能不良患者，有效率为100％。Cuadrado等[11]报告的62例患者中47例（75.8％）有效；Ghobadi等[12]报告的26例患者中16例获得完全缓解，5例获得部分缓解。Shahzad等[14]发表的综述和Meta分析纳入7篇文献共209例患者，显示纯化CD34^+^细胞输注的完全缓解率为72％（95％ *CI* 63％～79％）。本组8例患者中5例有效，纯化CD34^+^细胞输注后中位血象恢复时间为22（16～85）d，Ghobadi等[12]、Stasia等[15]、Klyuchnikov等[19]报道的中位起效时间分别为54（17～242）d、101（13～994）d、30（14～120）d。

影响纯化CD34^+^细胞输注疗效的因素尚不明确。Stasia等[15]报告了CD34^+^细胞输注治疗41例原发PGF的研究结果，并未发现造血恢复和患者临床特点（包括年龄、性别、疾病类型、疾病状态等）之间的相关性，且CD34^+^细胞输注数量对于造血功能恢复也无影响（*P*＝0.06）。Klyuchnikov等[19]发现，CD34^+^细胞输注治疗同胞供者与无关供者造血干细胞移植后PGF的有效率差异无统计学意义，造血恢复与原发病、供者类型、供患者年龄、供患者性别、供患者血型和CMV血症均无相关性。Cuadrado等[11]在CD34^+^细胞输注治疗62例PGF患者的研究中，供者类型、ABO血型不合、年龄、既往或活动性急/慢性GVHD、输注CD34^+^细胞数量、CMV再激活、输注CD34^+^细胞间隔等对造血重建没有影响，与供者性别匹配的患者具有较高的血象恢复率（90％对60％，*P*＝0.008），输注CD34^+^细胞期间是否合并感染对于血象恢复具有显著影响［92％（33/36）对50％（12/24），*P*<0.001］。

本研究有效的5例患者中均未发生急性GVHD，仅有2例发生慢性GVHD。Haen等[17]、Askaa[18]、Klyuchnikov等[19]报道CD34^+^细胞输注后急性GVHD发生率分别为5％、22％、19％。不同文章报道的GVHD发生率稍有差别，推测和输注物中CD3^+^细胞数有关[14],[23]。

本组8例难治性PHR患者接受纯化供者CD34^+^细胞输注后5例造血恢复，无输注相关急性GVHD发生，2例发生慢性GVHD，提示纯化供者CD34^+^细胞输注是治疗难治性PHR的有效方法。本项回顾性研究病例数较少，以上结论尚需继续深入研究加以验证。
